# A Clinical Communication Tool (Loop) for Team-Based Care in Pediatric and Adult Care Settings: Hybrid Mixed Methods Implementation Study

**DOI:** 10.2196/25505

**Published:** 2021-03-03

**Authors:** Amna Husain, Eyal Cohen, Raluca Dubrowski, Trevor Jamieson, Allison Miyoshi Kurahashi, Bhadra Lokuge, Adam Rapoport, Stephanie Saunders, Elaine Stasiulis, Jennifer Stinson, Saranjah Subramaniam, Pete Wegier, Melanie Barwick

**Affiliations:** 1 Temmy Latner Centre for Palliative Care Sinai Health Toronto, ON Canada; 2 Lunenfeld-Tanenbaum Research Institute Toronto, ON Canada; 3 Division of Palliative Care Department of Family and Community Medicine, University of Toronto Toronto, ON Canada; 4 Pediatric Medicine and Child Health Evaluative Sciences The Hospital for Sick Children Toronto, ON Canada; 5 Child Health Evaluative Sciences, Research Institute The Hospital for Sick Children Toronto, ON Canada; 6 Department of Medicine Unity Health Toronto Toronto, ON Canada; 7 Pediatric Advanced Care Team The Hospital for Sick Children Toronto, ON Canada; 8 Emily's House Children's Hospice Toronto, ON Canada; 9 School of Rehabilitation Sciences Faculty of Health Sciences McMaster University Hamilton, ON Canada; 10 Institute of Medical Science University of Toronto Toronto, ON Canada; 11 Humber River Hospital Toronto, ON Canada; 12 Department of Psychiatry Faculty of Medicine University of Toronto Toronto, ON Canada; 13 Dalla Lana School of Public Health University of Toronto Toronto, ON Canada

**Keywords:** coordination of care, complexity, internet communication technology, collaborative care, implementation science, theory of behavior, interprofessional team, patient engagement, social networking technology, user-centered design, Consolidated Framework for Implementation Research, Quality Improvement Framework, Implementation Outcome Taxonomy

## Abstract

**Background:**

Communication within the circle of care is central to coordinated, safe, and effective care; yet patients, caregivers, and health care providers often experience poor communication and fragmented care. Through a sequential program of research, the Loop Research Collaborative developed a web-based, asynchronous clinical communication system for team-based care. Loop assembles the circle of care centered on a patient, in private networking spaces called Patient Loops. The patient, their caregiver, or both are part of the Patient Loop. The communication is threaded, it can be filtered and sorted in multiple ways, it is securely stored, and can be exported for upload to a medical record.

**Objective:**

The objective of this study was to implement and evaluate Loop. The study reporting adheres to the Standards for Reporting Implementation Research.

**Methods:**

The study was a hybrid type II mixed methods design to simultaneously evaluate Loop’s clinical and implementation effectiveness, and implementation barriers and facilitators in 6 health care sites. Data included monthly user check-in interviews and bimonthly surveys to capture patient or caregiver experience of continuity of care, in-depth interviews to explore barriers and facilitators based on the Consolidated Framework for Implementation Research (CFIR), and Loop usage extracted directly from the Loop system.

**Results:**

We recruited 25 initiating health care providers across 6 sites who then identified patients or caregivers for recruitment. Of 147 patient or caregiver participants who were assessed and met screening criteria, 57 consented and 52 were enrolled on Loop, creating 52 Patient Loops. Across all Patient Loops, 96 additional health care providers consented to join the Loop teams. Loop usage was followed for up to 8 months. The median number of messages exchanged per team was 1 (range 0-28). The monthly check-in and CFIR interviews showed that although participants acknowledged that Loop could potentially fill a gap, existing modes of communication, workflows, incentives, and the lack of integration with the hospital electronic medical records and patient portals were barriers to its adoption. While participants acknowledged Loop’s potential value for engaging the patient and caregiver, and for improving communication within the patient’s circle of care, Loop’s relative advantage was not realized during the study and there was insufficient tension for change. Missing data limited the analysis of continuity of care.

**Conclusions:**

Fundamental structural and implementation challenges persist toward realizing Loop’s potential as a shared system of asynchronous communication. Barriers include health information system integration; system, organizational, and individual tension for change; and a fee structure for health care provider compensation for asynchronous communication.

## Introduction

### Background

Collaboration is fundamental to the care of patients with complex needs [1]. Optimal patient outcomes require integrated cross-disciplinary expertise alongside patient and caregiver engagement [2,3]. Effective communication within the circle of care is essential for coordination, cooperation, collaboration, safety, quality, and cost-effectiveness; yet poor communication and fragmented care is too often the norm [2,3].

In this paper, we use the concepts of communication, coordination, cooperation, and collaboration as defined by Fuks et al [4] and as employed in Eikey et al’s [5] review of health information technologies and collaboration. Communication is the “exchange of messages and information among people; coordination is the management of people, their activities and resources; cooperation is the production taking place on a shared workspace.” Collaboration encompasses communication, coordination, and cooperation, but is much more than its parts [4,5]. Collaboration includes “the development and testing of rules of engagement and shared understanding that facilitates how people work together” [4,5].

A key element of quality care is that patients and families experience good continuity of care (COC); care that is connected and coherent over time [6,7]. Because informational, management, and relational continuity are aspects of COC, effective communication among team members is likely to improve the patient-level outcome of COC.

To promote effective communication, and to examine how this relates to other aspects of collaboration and COC, we developed Loop, a web-based, asynchronous clinical communication tool formerly called My Team of Care (myTOC) [8]. Loop enables private threaded communication among patients, their caregivers, and their health care providers. In this paper, “teams” refers to the members of these digital circles of care called “Patient Loops.”

Loop emerged from the need to facilitate open communication between all members of the circle of care, regardless of their role, organizational affiliation, or geographic location. Loop was developed using user-centered design principles. The interface is intuitive [9], allowing patients and caregivers to communicate with their various health care providers (HCPs) in the Patient Loop in a flexible and timely way [10].

To date, although similar tools have been developed and taken to market [11-14], no such tool has been successfully implemented at scale. This raises the question of whether communication tools such as Loop are useful and, if so, what factors might impact their implementation and clinical effectiveness and ultimate scalability.

A pilot randomized controlled trial (RCT) of Loop in patients with advanced cancer demonstrated that Loop was intuitive and usable by members of the patient care team and used as intended for team-based communication in some Patient Loops. There was a nonsignificant trend in improved patient self-reported COC in the intervention group over the 3-month study period [8]. Adoption was influenced by a complex set of system, organizational, team, and individual factors, which is consistent with evidence on determining factors associated with effective implementation [15]. This study examines implementation barriers or facilitators, while also exploring Loop’s clinical and implementation effectiveness.

### Implementation of eHealth Technologies

eHealth is defined as the application of information, computer, or communication technology to some aspects of health or health care [16]. The widespread use and integration of eHealth interventions into routine care remains a challenge, and most eHealth technologies linger within the confines of the academic settings in which they are studied and are not sustained in practice [17]. Implementation science can address this problem by studying contextual factors [18], process [19], and intervention effects that result in eHealth technologies that are more externally valid, practical, and sustainable, while identifying issues that are important to stakeholders and users.

A theory-informed approach to studying eHealth technology implementation addresses weaknesses reported in existing studies, namely, that they are often based on one particular technology, setting, or health condition, making it difficult to access the available evidence that can inform implementation planning [16]. A recent systematic review of 37 eHealth technologies analyzed using the Consolidated Framework for Implementation Research (CFIR) [15] as an organizing framework recommended that eHealth technology implementation should consider the following highly salient factors: complexity, adaptability, compatibility, cost, and champions. Identifying and monitoring of these barriers can support implementation planning, inform the use of mitigating implementation strategies, and improve implementation effectiveness [20].

### Implementation Frameworks

As there is no implementation science model that specifically addresses eHealth technology, we utilized well-established models of implementation to inform the process, factors, and outcomes for this study. The Quality Improvement Framework (QIF) [21] was derived from 25 implementation process frameworks to foster high-quality implementation. The QIF lays out 4 phases that serve as a useful blueprint for the implementation process: phase 1, initial considerations for readiness in the host setting; phase 2, creating a structure for implementation; phase 3, offering the intervention and monitoring ongoing structure; phase 4, sustaining the practice and improving future applications.

The CFIR [15] is a determinant framework comprising 39 key factors associated with successful implementation, structured within 5 domains: intervention characteristics, inner setting, outer setting, characteristics of individuals, and the implementation process. Recent research by Barwick and others [22,23] has identified a subset of factors found to be more salient across contexts. This knowledge can streamline the assessment of barriers toward more effective implementation.

Implementation outcomes [24] are distinct from clinical outcomes and capture effects of deliberate actions to implement interventions in new settings. Implementation outcomes have 3 important functions: (1) they serve as indicators of implementation success; (2) are proximal indicators of implementation processes; and (3) are key intermediate outcomes in relation to clinical outcomes. Implementation outcomes include acceptability, adoption, appropriateness, cost, feasibility, fidelity, penetration, and sustainability. When interventions fail to produce desired outcomes, it is important to know if the failure occurred because the intervention was ineffective (intervention failure) or whether it was implemented incorrectly (implementation failure).

### Objectives

The study examined the implementation and clinical effectiveness of Loop across 6 health care settings. We assessed clinical outcomes (COC, client participation in decision- making), implementation outcomes (adoption, acceptability, appropriateness, and feasibility), and explored implementation barriers and facilitators. We hypothesized that an implementation approach informed by the core principles of implementation science (ie, process, factors, strategies, outcomes, and implementation team) would lead to adoption, and that higher Loop use would be associated with improved patient COC. We anticipated identifying similar salient determinant factors that have been documented across varied study contexts and interventions. The study reporting adheres to the Standards for Reporting Implementation Research (Multimedia Appendix 1) [25].

## Methods

### Study Design

The study design was a hybrid type II, involving the simultaneous testing of a clinical intervention and an implementation strategy with the aim of more rapid translation [26]. We used a mixed methods approach to examine the clinical and implementation effectiveness of Loop at 6 health care sites [26]. The study was approved by the Research Ethics Board of Sinai Health System, University Health Network, and SickKids Hospital, and was conducted in Toronto, Ontario, Canada, where health care is provincially funded.

### Loop Intervention

**Figure 1:**
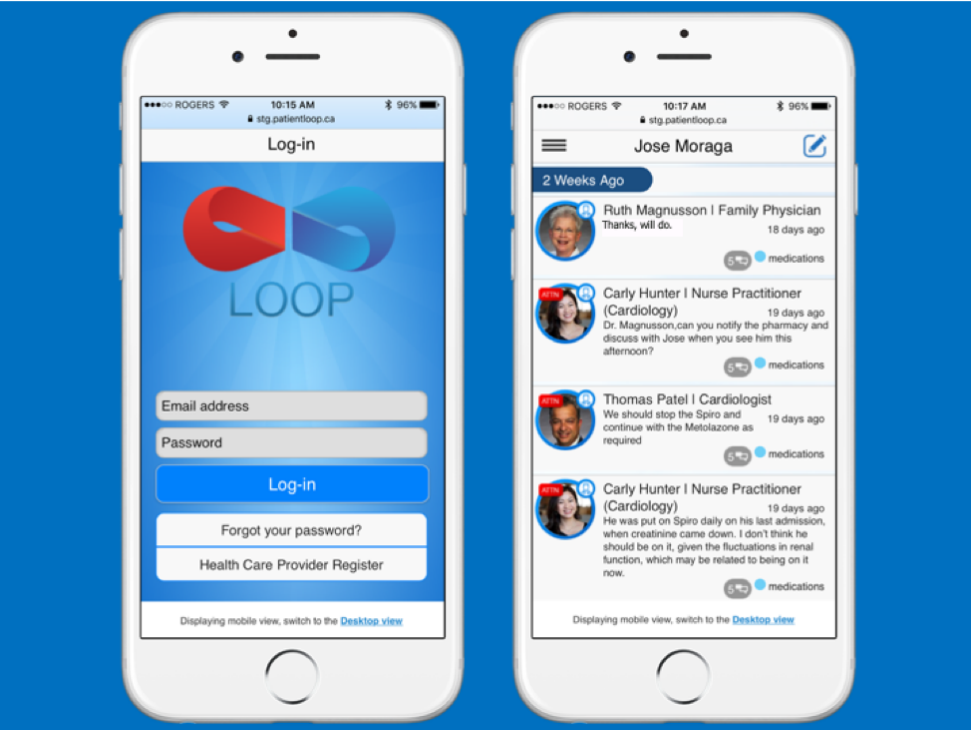
Screenshots of Loop optimized for a smartphone.

**Figure 2:**
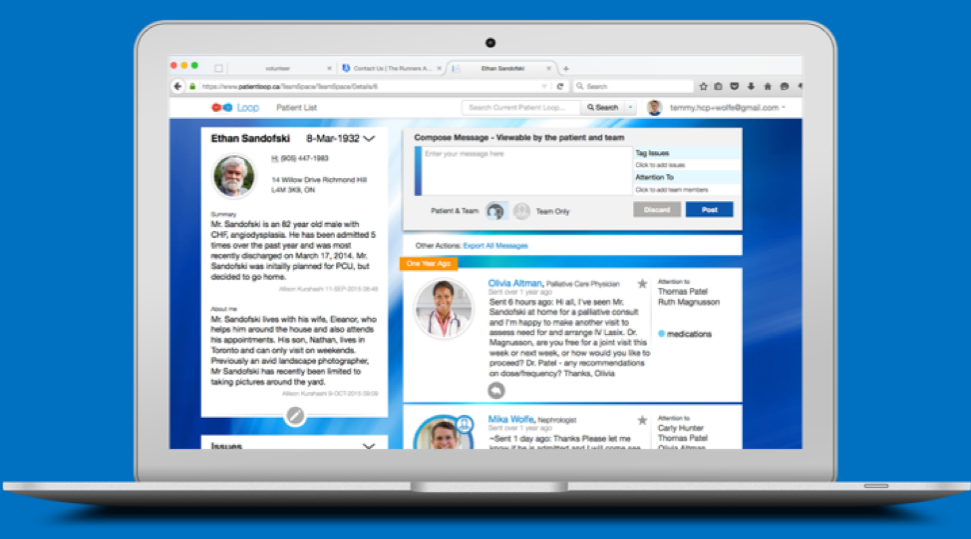
Screenshot of Loop on a computer screen.

**Figure 3:**
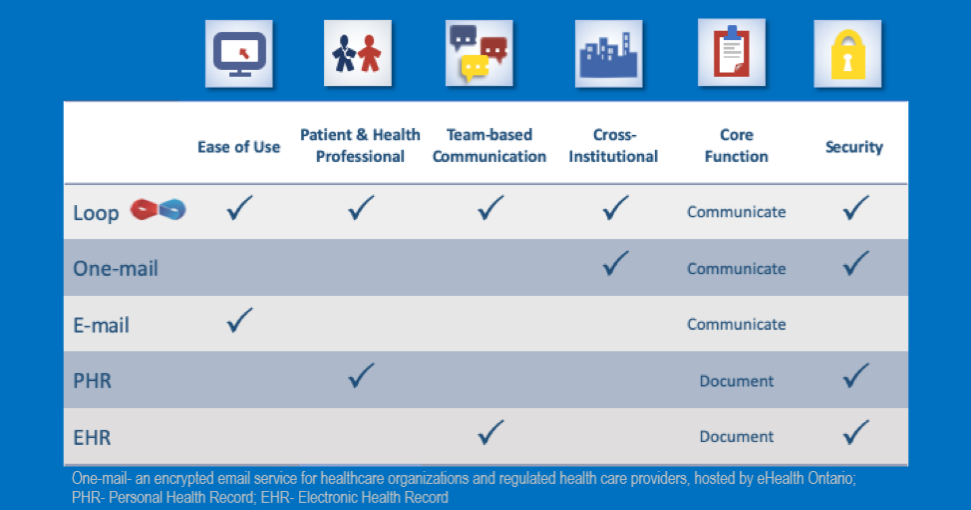
Comparison of Loop with other categories of eHealth tools.

Loop enables private communication groups centered on a patient, called Patient Loops (Figures 1 and 2). In each Patient Loop, there are 2 streams of communication, one that includes the patient and caregiver, and another that is for the health care providers only [27]. Messages are threaded for ease of viewing conversations. Messages may be tagged with customizable labels (eg, hypertension, pain, lymphedema), and marked to the attention of a specific member or members of the Patient Loop. The latter action triggers a deidentified link to be sent to the email of the intended recipient(s) [28]. Figures 1 and 2 depict the Loop interface, and Figure 3 shows how Loop functionality compares to other categories of eHealth tools.

### Site Recruitment

Six clinical sites participated in this study. All sites were in academic health organizations in Toronto, Ontario, Canada. Three clinical sites were recruited in the first roll-out, including a regional palliative care program (Site 1) that provides home-based palliative care, alongside home care organizations; an academic family health program (Site 2) that provides primary care to patients; and a brain metastases clinic (Site 3) housed within an outpatient regional cancer center. These first 3 sites were approached during the knowledge translation activities for the previous stages of the research program. For this study, we reached out to health care colleagues in various specialties to present the study aims to site leads. Once site leads expressed interest in participating, we presented to the broader clinical group at each site.

In a second roll-out, we recruited a pediatric blood and marrow transplant (Site 4) program, and a pediatric palliative care program (Site 5)—both pediatric sites are situated within the same quaternary pediatric teaching hospital; and an outpatient psychosocial oncology program (Site 6) at a regional cancer center. The sites recruited in this second roll-out approached us, having learned of the study from colleagues or the Loop Research Collaborative. Five of the sites were specialized in hematology-oncology, radiation-surgical oncology, psychosocial oncology, or palliative care. The family medicine program was included to examine Loop adoption in primary care. An implementation champion was identified at each site. All champions were clinicians, most had a leadership role, and they engaged other HCPs at their site to elicit participation in the study.

### Recruitment of HCPs, Patients, and Caregivers

The implementation champion at each site identified initiating HCPs (iHCPs) for recruitment. Additionally, study staff identified and recruited iHCPs at implementation planning activities described below. iHCPs then identified patients or their caregivers who were screened for inclusion criteria. Once the iHCP and the patient were registered in Loop, both were asked to identify any additional HCPs from the patient’s circle of care who could be invited to participate on the Loop. Study staff or iHCPs invited additional HCPs via email, phone, and in-person. There was no limit to the number of additional HCPs invited to join the patient’s Loop, and all provided verbal consent upon joining. Study staff followed a standard procedure to invite and enroll participants. The Loop Help menu contains videos for a Loop “quick start” guide.

### Inclusion Criteria

Patients/caregivers from adult centers were included if (1) they were aged 18 or older and had capacity to consent. Pediatric patients (18 years or younger) could consent themselves, if capable, otherwise their parent or guardian was consented; (2) the patient or caregiver had internet access; and (3) there were at least two HCPs involved in the patient’s care. An additional criterion for patients recruited from adult centers was an Eastern Cooperative Oncology Group (ECOG) Performance Status score of 2 or less [29,30]. There was no comparable performance status measure for pediatric patients.

### Exclusion Criteria

Patients were excluded if they (1) they had a prognosis of less than 6 months as determined by a physician, except for adult and pediatric palliative care sites where it was difficult for clinicians to identify those that met this criterion; or (2) had cognitive impairment as determined by a physician or by study staff using the Bedside Confusion Scale for adult patients [31].

### Sample Size

No sample size calculation for clinical effectiveness was possible due to the limited sample size of the feasibility trial. Based on the previous study in ambulatory cancer and palliative care [8], we anticipated it would be feasible to recruit 15 teams during the first wave of recruitment at 3 sites and 5 teams from each additional site during the second wave, resulting in 60 teams or Patient Loops. Based on a 25% attrition rate at various steps of enrollment, onboarding, and assembling the team, we anticipated a total enrollment of 45 Patient Loops in this study.

### Implementation Procedure

The initiating context for this implementation endeavor was research. The intention was to provide Loop to participating organizations with the aim of exploring implementation and clinical effectiveness. We did not set out to implement Loop within entire organizations. As such, we did not undertake certain implementation activities such as developing organizational implementation teams and ensuring sustainability, as these are process elements key to program- or organizational-level implementation. Previous research [32] has identified that initiating context or impetus for the implementation endeavor is important for implementation process and sustainability.

#### Implementation Phase 1

Phase 1 occurred over 3-6 months and focused on understanding the initial implementation considerations within each site (QIF Phase 1) [21]. An assessment of needs, capacity, and readiness was done at each site, led by study staff, and guided by the Hexagon Tool [33]. The purpose of these meetings was to explore process adaptations that might be required, clarify goals, provide information about collaborative care and Loop, and to establish buy-in for using Loop. We conducted workflow observations to understand HCPs’ clinical workflows. Focus groups and consultative meetings were held to introduce and refine a tailored implementation plan for the study. HCPs at each initiating site were invited to participate in the information meetings where they were recruited as iHCPs, registered on Loop, and baseline data were collected.

#### Implementation Phase 2

Phase 2 spanned 3-6 months and focused on infrastructure and workflow adjustments for implementing Loop (QIF Phase 2) [21]. Using the description drawn from the implementation framework, site implementation champions were identified among HCPs who expressed interest in this role, although no formal role designation was made at the program level. Site readiness assessments from phase 1 informed a general implementation plan for each site, which was discussed with site champions and HCPs for refinement. iHCPs at all sites were asked to identify patients who met inclusion criteria. Patients or, if appropriate, their caregivers were then consented, enrolled in the study, and registered on Loop, creating a Patient Loop. Patient participants could identify a caregiver to participate in the study, who was also consented and joined the Patient Loop. Patients and their iHCPs were asked to identify other members of the patient’s circle of care (additional HCPs) who were invited to join the Patient Loop and were verbally consented. At the time of registration on Loop, study staff showed new Loop users how to use Loop.

#### Implementation Phase 3

Phase 3 focused on initiating Loop use at the site and providing ongoing supports [21]. Loop was available to each site and team for up to 8 months, during which data were collected. Implementation strategies were used to maintain user engagement, including (1) monthly check-in phone calls with participants to gather information on user experiences, provide support, and troubleshoot Loop use; and (2) periodic audit and feedback summaries on Loop uptake posted by study staff in each Patient Loop at bimonthly intervals. Feedback summaries were intended to remind users to use Loop and as a positive peer pressure stimulus to encourage Loop use.

#### Implementation Phase 4

For this study, Phase 4 involved an ongoing process of reflection on future applications for Loop [21] and was concurrent with all phases. Study participants, implementers, and stakeholders reflected on implementation process, Loop use, and improvements. These reflections were captured during regular interviews and periodic stakeholder consultations.

### Data Collection

#### Participant Characteristics

Site characteristics were gathered during Phase 1 activities and culminated in an implementation plan for each site. Baseline data for participant characteristics included demographics, internet preferences, performance status (Palliative Performance Scale) [34], and Age-Adjusted Charlson Comorbidity Index (ACCI) [35] for adult patient participants. In a sample of cancer patients, ACCI scores have been categorized as mild (0-1), moderate (2-3), and severe (≥4), corresponding to a significant difference in survival rates [35]. For patients recruited from adult centers, the iHCP determined if patients had high unmet health or social needs as defined by Schaink et al [36]. Although a Pediatric Comorbidity Index is being developed, there is currently no validated measure of comorbidity or complexity for pediatric patients [37].

#### Implementation Outcomes

##### Adoption

Adoption is defined as the intention, initial decision, or action to try or employ an intervention or evidence-based practice [24]. In this study, adoption was operationalized as a function of Loop use: (1) the number of patient care teams registered on Loop, (2) the number of participants in each user category on Loop, (3) the total number of messages by site, and (4) the median number of messages per team per site. Loop use metrics were collected from Loop software reporting and backend data export at an interim point and at the end of the study, and by participant self-report in the check-in interviews.

##### Acceptability

Acceptability is the perception among implementation stakeholders that a given treatment, service, practice, or innovation is agreeable, palatable, or satisfactory [24]. This was assessed informally in the phase 1 preparatory meetings and within the CFIR interviews.

##### Appropriateness

Appropriateness is the perceived fit, relevance, or compatibility of the intervention for a given practice setting, provider, or consumer; or perceived fit of the innovation to address a particular issue or problem [24]. This was assessed informally in the phase 1 preparatory meetings and in the CFIR interviews and monthly check-ins.

##### Feasibility

Feasibility is defined as the extent to which a new intervention can be successfully used or carried out within a given setting [24]. The feasibility of implementing Loop was assessed at the site level with respect to number of sites approached; number of sites who approached us with an interest in participating; number of HCPs interested and recruited; number of HCPs who identified patients for recruitment; number of patients, caregivers, and additional HCPs recruited; and number of active Patient Loops assembled.

Cost, fidelity, penetration, and sustainability were not measured in this study. Fidelity to the intervention, or the extent to which users adhered to the Loop tool as intended [24], did not apply because as an eHealth technology, Loop does not have optional multiple core components; rather, a message is sent or not. Penetration and sustainability [24] were not relevant because Loop was only made available to a discrete number of teams for the purpose of this study, and there was no intention of full implementation within each site as part of usual practice.

#### Barriers and Facilitators

Barriers and facilitators to Loop implementation were assessed using individual interviews informed by implementation outcomes [24] and CFIR [15] using 2 main qualitative approaches. A brief (5-20 min) monthly check-in phone call with patients, caregivers, and iHCPs was used to gather feedback and troubleshoot implementation and technical issues. Participants were asked about their Loop use over the previous month, and their perception of its acceptability, accessibility, usefulness, feasibility, including the impact of Loop use on workflow, and their willingness to continue using Loop beyond the study if given the opportunity. Study staff conducted and captured monthly check-in content in fieldnotes. Check-in phone calls were audio recorded and reviewed to support fieldnote rigor.

Semistructured interviews based on the CFIR and adapted for language and context were conducted by telephone to capture HCP perspectives on implementation barriers and facilitators (Multimedia Appendix 2). All CFIR domains and constructs were included except for trialability, as this factor did not apply in a research-initiated implementation endeavor. In addition, given the role of patients in the use of Patient Loops, we included a sixth domain related to Patient Beliefs and Experiences to capture HCP perspectives on how patients experienced Loop, which has been done in previous studies [23]. The interview protocol was piloted with 2 HCPs and revised for length, flow, and clarity. Interviews were conducted with the site lead and an additional iHCP from each site who were purposefully sampled to capture sites having higher and lower Loop use. CFIR interviews were 30-60 minutes long, conducted by 2 members of the research team experienced in CFIR interviews and analysis (ES and RD), and supervised by the implementation science lead (MB).

#### Clinical Outcomes

Clinical outcomes were collected at baseline and at 2-month intervals from all patients or caregivers, either by phone or in person using standardized measures administered via survey (Table 1). Measures assessed patient or caregiver experience of COC, symptom severity, and participation in decision making and goal setting. Internally developed questionnaires measured team effectiveness [38]. Details on circle of care communications occurring outside the Patient Loop were collected from iHCPs, patients, and caregivers at monthly intervals using an internally developed social network questionnaire and will be the focus of a separate paper. Clinical effectiveness outcome measures were not collected from the noninitiating (additional) HCPs to decrease respondent burden and encourage enrollment on Patient Loops.

**Table 1:** Patient and caregiver outcome measures.

Construct	Survey	Validated in	Scoring details	Administered to
Continuity of care experience (COC)	Continuity and Coordination subscale, Picker Ambulatory Cancer Care Scale [7]	Patients with cancer	Range: 0-100 Higher scores indicate higher continuity of care	Adult and pediatric patients and their caregivers
Symptom severity	Edmonton Symptom Assessment Scale (ESAS) [39,40]	Adult patients with cancer	Range: 0-90 Higher scores indicate higher symptom severity	Adult patients
Symptom bother	Symptom Screening in Pediatrics Tool (SSpedi) [41,42]	Children with cancer and hematopoietic stem cell transplantation	Range: 0-60 Higher scores indicate higher levels of bother	Pediatric patients and their caregivers
Client participation in decision making and goal setting (CPDG)^a^	Client-Centered Rehabilitation Questionnaire (CCRQ) [43], CPDG domain	Discharged rehabilitation patients	Range: 0-100 Higher scores indicate more positive responses	Adult and pediatric patients and their caregivers

^a^CPDG: Client participation in decision-making and goal setting domain of Client Centered Rehabilitation Questionnaire (CCRQ).

### Analysis of Participant Characteristics

Participant characteristics (patients, caregivers, and HCPs) are described by site using frequencies, medians or means, SDs, and ranges.

### Analysis of Implementation Outcomes

#### Adoption

Loop adoption was dependent on the number of individuals in each Patient Loop. A Patient Loop was considered active if it included an iHCP and a patient or caregiver. We conducted a subanalysis of the proportion of Loops with at least one additional HCP as part of the care team assembled on Loop.

#### Monthly Check-in Interviews (Adoption, Acceptability, Appropriateness, Barriers, and Facilitators)

Monthly check-in data were analyzed using hybrid data-theory-driven content analysis [44] on MAXQDA 2018.2. An initial codebook based on monthly check-in questions was developed and iteratively revised throughout the analytic process. The first phase of coding involved 6 members of the project team (AH, MB, PW, StS, SaS, and BL) who independently coded the same 3 monthly check-in interview notes, followed by a discussion to achieve consensus and identify revisions to the codebook. Each coder then rated the same notes from 3 new interviews, which were again reviewed for consensus and codebook revisions. The 6 coders continued coding the remainder of the interview notes independently and met regularly to discuss any new codes and issues related to implementation. Following coding completion, 1 coder (AH) reviewed monthly check-in notes from all sites, identified common coding themes, and summarized excerpts using data-driven content analysis [45]. In addition, some divergent perspectives were analyzed to provide a range of perspectives. Excerpts and summaries were discussed by the 6 coders to achieve agreement on emergent summative statements. The main coder (AH) then synthesized summative statements from text segments within categories and further sorted and analyzed all coded segments by site. A second reviewer (MB) reviewed the summary tables, consisting of coding categories, exemplar excerpts from the interview notes, and summative statements by site.

#### CFIR Interviews (Acceptability and Appropriateness)

CFIR interviews were analyzed using an adapted rapid analysis method [46]. ES conducted the interview while RD simultaneously coded each interview against CFIR constructs using a pre-set template. After each interview, ES checked her notes against the audio recording for completeness, and then RD sent her coded notes to ES to do a final check for accuracy and completeness. In a second step, not part of the rapid analysis method but consistent with previous CFIR research [47], ES and RD independently assigned valence ratings to each construct based on its strength (–2, –1, 0, 1, 2) and direction (negative or positive) relative to Loop implementation. Disagreements on valence ratings were resolved by discussion and consensus with MB.

Implementation barriers and facilitators were compared within and across sites and CFIR constructs were explored as a function of high and low Loop use. Data overlap between CFIR constructs and monthly check-in data were explored using a mixed methods approach to achieve greater contextual understanding [48].

### Analysis of Clinical Effectiveness Outcomes

We report descriptive statistics (means, SD, and ranges) for clinical outcome measures. We conducted an analysis of change in score of the main outcome of the Continuity and Coordination Subscale of the Picker (COC) between baseline and each timepoint. We did an exploratory repeated measures analysis of COC controlling for patient participation in decision making (CPDG domain of Client-Centered Rehabilitation Questionnaire [CCRQ]) and Loop use within the Patient’s Loop.

## Results

### Recruitment

**Figure 4:**
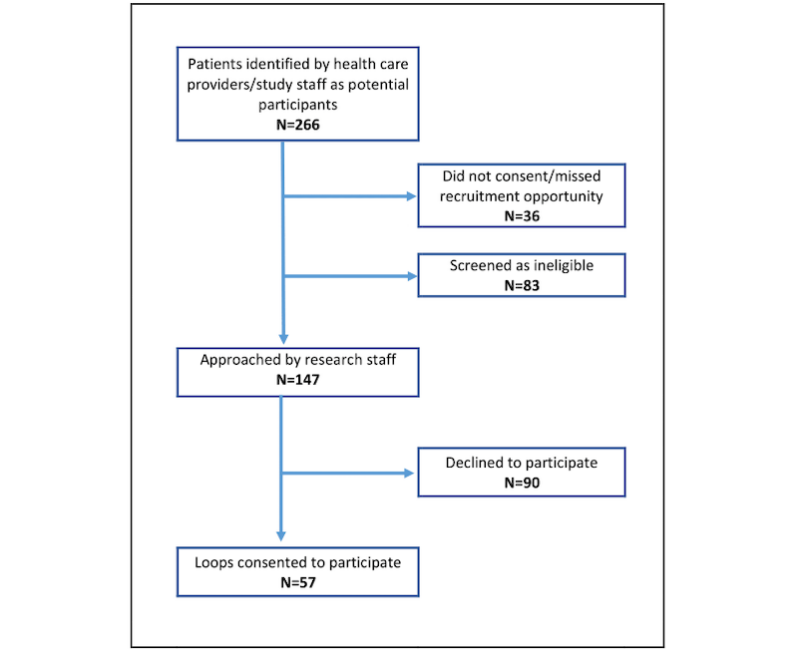
Patient recruitment flowchart.

A total of 57 HCPs and key informants took part in Phase 1 activities. We recruited 25 iHCPs across all sites who then identified 266 patients as potentially meeting participation criteria. Figure 4 charts the steps in participant recruitment (patients or caregivers) from which Loops were formed.

Of the 147 patient participants (or caregivers in their lieu) who were assessed and met screening criteria, 57 consented and 55 Loops were created, within which 51 patients participated in data collection. Of the 55 Loops created, attrition resulted in 31 Loops completing the follow-up period. Patients and iHCPs together identified 190 unique additional HCPs who were part of the patient’s circle of care (some HCPs were included in more than 1 Patient Loop). Research staff contacted each identified additional HCP an average of 4 times, using phone and email, to invite them to participate in the study. Of these individuals, 96 (50.5%) consented to join a Loop. Of the remaining additional HCPs, 47/190 (24.7%) did not respond to invitations by the research team, 30/190 (15.8%) declined to join, and 17/190 (8.9%) were unable to participate further in the study as the referent patient had died. We did not monitor reasons for declining to join.

### Participant Characteristics

Baseline participant characteristics are presented in Tables 2 and 3. Of 51 patients for whom baseline data were collected, 59% (30/51) were female. Patients ranged in age from 1.4 to 90 years. Most patients had a cancer diagnosis (61%, 31/51), although the primary diagnoses ranged from pediatric genetic disorders to connective tissue diseases. For adult patients, performance status was collected with the Palliative Performance Scale, for which median scores ranged from 60% at Site 1 to 80% at Site 6. The minimum Palliative Performance Scale score was 50% and the maximum score was 100% across all sites. The median ACCI comorbidity score ranged from mild in primary care patients to severe among home palliative care patients, demonstrating variable morbidity–mortality within the sample.

**Table 2:** Patient and caregiver characteristics.

Characteristics			Site 1	Site 2	Site 3	Site 4	Site 5	Site 6	Overall sample
**Participant, n**									
	Patient		6	10	16	3	5	11	51
	Caregiver		2	0	4	1	5	3	15
**Gender, n**									
	**Patient, n**								
		Female	3	7	11	0	2	7	30
		Male	3	3	5	3	3	3	20
		Other						1	1
	**Caregiver, n**								
		Female	1	—	4	—	—	0	5
		Male	1	—	0	—	—	3	4
**Age (years), median (range)**									
	Patient		68 (58-90)	62 (18-87)	58 (29-73)	17 (11-27)	14.5 (1.4-17)	52 (25-72)	
	Caregiver		73 (67-79)	—	39.5 (38-60)	—	—	65 (54-66)	
Performance status, median % (range)			60 (50-80)	90 (60-100)	80 (60-100)	—	—	80 (70-100)	
**Patient diagnoses, n**			6	10	16	3	5	11	51
	Cancer		6	1	13	0	4	7	31
	Noncancer		0	9	3	3	1	4	20
**Age-Adjusted Charlson Comorbidity Index (ACCI), median (range)**			6 (3-8)	3 (0-9)	5 (0-9)	—	—	2 (0-6)	
	Severity (median)		Severe	Mild	Severe	—	—	Mild	
**Complexity, n**			2	5	15	—	—	9	31
	Multimorbidity		2	5	13	—	—	7	27
	Resource utilization		2	2	12	—	—	6	22
	Psychosocial issues		1	1	2	—	—	9	13

**Table 3:** Health care provider characteristics.

Characteristics		Site 1	Site 2	Site 3	Site 4	Site 5	Site 6	Overall sample
**Health care provider, n**								
	Initiating HCP^a^ (iHCP)	6	6	4	2	3	4	25
	Additional HCP	11	14	26	6	19	20	96
**iHCP gender**								
	Female	2	3	1	1	2	3	12
	Male	4	3	3	1	1	1	27
iHCP age (years), median (range)		41 (30-68)	39 (32-67)	40.5 (32-44)	62 (62)	37 (36-39)	49.5 (33-54)	
**iHCP type, n**								
	Physician	6	5	4	2	—	4	21
	Clinical nurse specialist (CNS)	—	—	—	—	2	—	2
	Nurse practitioner (NP)	—	1	—	—	1	—	2
**Clinical specialty**		Focused palliative care practice (N=6)	Family physician (N=5); NP (N=1)	Radiation oncology (N=3); Neurosurgeon (N=1)	Pediatric hematologist/oncologist (N=2)	Palliative CNS (N=2); palliative NP (N=1)	Psychiatry (N=4)	
**Role, n**								
	Administrative director	—	1	—	1	—	—	2
	Clinical programs director	2	3	1	—	—	1	7
	Clinical care	6	6	4	2	3	4	25
Years in health care, median (range)		10.5 (3-37)	12.5 (4-38)	14.5 (6-18)	28 (24-32)	13 (11-15)	15 (6-19)	
**Practice fee structure**								
	Fee for service	—	1	1	—	—	4	6
	Alternate payment plan	6	3	—	1	—	—	10
	Salaried	1	2	2	1	3	—	9
	Other, academic, or alternate funding plan	—	Capitated alternate payment plan (n=3)	Fee for service/alternate funding plan (n=1)	—	—	—	—

^a^HCP: health care provider.

For 4 of 6 sites (Sites 1, 2, 3, and 6), iHCPs were asked to assess patient complexity based on the categories identified by Schaink et al [36]. The majority of patients were identified as having multimorbidity (87%, 27/31) and high resource utilization (71%, 22/31), while a minority were identified as having psychosocial issues (42%, 13/31).

Although internet access was an inclusion criterion, 1 out of 51 patient reported no internet access. Almost all patients and caregivers reported feeling “comfortable” or “very comfortable” using computers, and most felt “comfortable or very comfortable” using a smartphone. Of 51 patients or their caregivers, 24 (47%) used email and 16 (31%) used text to communicate with their HCPs; the remainder communicated by phone, in-person, or by pager.

### Implementation Outcomes

#### Adoption

Loop adoption was based on use statistics pulled from Loop’s data server. Across all sites, the total number of participants, including additional HCPs, who joined Patient Loops was 262, with 52 Patient Loops created. The median number of HCPs per Patient Loop was 4 with a range of 1-13 (Table 4). Overall, 228 Loop messages were sent by patients, caregivers, and health care providers within the study period. A full breakdown of messages sent by user type and site is presented in Table 5. Across sites, a median of 1 message and a maximum of 28 messages were sent within a single team. Quartiles were calculated to characterize the number of messages as low, medium, and high Loop use based on the number of messages exchanged at each site: Q1 (low), Q2 (medium), and Q3 (high). Patients were the most active users, posting nearly 50% of all messages within Loop.

**Table 4:** Team composition by Patient Loop (team) and by site.

Composition			Site 1^a^	Site 2^b^	Site 3^c^	Site 4^d^	Site 5^e^	Site 6^f^	All sites
Members^g^, n			26	27	109	14	49	37	262
**Teams, n**			6	9	18	3	5	11	52
	**HCP^h^ per team**								
		Mean (SD)	3.17 (2.14)	2.00 (1.00)	4.89 (0.96)	3.33 (1.15)	7.80 (4.32)	2.09 (1.22)	3.79 (2.44)
		Median (range)	2 (1-7)	2 (1-4)	5 (4-7)	4 (2-4)	7 (2-13)	2 (1-4)	4 (1-13)
		≥1 secondary HCP	5	5	18	3	5	7	43
	**Members per team**								
		Mean (SD)	4.33 (2.16)	3.00 (1.00)	6.06 (1.11)	4.67 (0.58)	9.80 (4.32)	3.36 (1.36)	5.04 (2.62)
		Median (range)	3 (2-8)	3 (2-5)	6 (5-8)	5 (4-5)	9 (4-15)	3 (2-5)	5 (2-15)

^a^Six initiating HCPs were recruited from among 18 physicians within an expert palliative care program that has a large homecare component.

^b^Six initiating HCPs were recruited from among 12 physicians in an academic family medicine site.

^c^Three radiation oncologists and 1 neurosurgeon (iHCPs) were recruited from within a multidisciplinary program based in a regional cancer center, which included additionally 2 neurosurgeons, 1 registered nurse (RN), 1 physician assistant (PA), and 1 fellow in training. The PA and Fellow participated as additional HCPs on the Patient Loops.

^d^Two out of 5 physicians, 4 patients, and 2 caregivers were recruited from a Pediatric Blood and Marrow Transplant program within a quaternary pediatric hospital. Additionally, this program has 3 nurse practitioners (NPs) and 4 RNs.

^e^One NP and 2 clinical nurse specialists were recruited as iHCPs from a pediatric palliative care program, which includes 5 physicians, 1 nurse practitioner, and 2 clinical nurse specialists within a quaternary pediatric hospital.

^f^Four out of 9 psychiatrists were recruited. Additionally, this adult psychosocial oncology program located within a regional cancer center has 16 social workers, 5 clinical psychologists, and 2 music/art therapists.

^g^Includes patient, caregiver, and HCP members.

^h^HCP: health care provider.

**Table 5:** Message frequency by site, user type, and Patient Loops (teams).

Message Frequency		Site 1	Site 2	Site 3	Site 4	Site 5	Site 6	All sites
**Messages (not including research administrator), N**		39	80	62	9	26	12	228
	Median (range)	3.5 (0-22)	3 (0-27)	1.5 (0-28)	4 (0-5)	1 (0-18)	0 (0-7)	1 (0-28)
	Frequency quartiles (Hi, Med, Lo)	Q3 (Hi)	Q3 (Hi)	Q3 (Hi)	Q1 (Lo)	Q2 (Med)	Q2 (Med)	
**Messages sent by user type, n (%)^a^**								
Total messages (including research administrator), N		54	98	97	14	30	25	318
	Patient	12 (22.2)	53 (54.1)	28 (28.9)	1 (7.1)	0 (0.0)	7 (28.0)	101 (31.8)
	Caregiver	18 (33.3)	0 (0.0)	14 (14.4)	3 (21.4)	14 (46.7)	1 (4.0)	50 (15.7)
	Health care provider	9 (16.7)	27 (27.6)	20 (20.6)	5 (35.7)	12 (40.0)	4 (16.0)	77 (24.2)
	Research admin	15 (27.8)	18 (18.4)	35 (36.1)	5 (35.7)	4 (13.3)	13 (52.00)	90 (28.3)
Teams ≥1 message, n		5	7	10	2	3	5	33

^a^The sum of all messages, including those sent by the Research Admin, were used as the denominator when calculating the % in this section.

Participants also reported their time spent on Loop in the monthly check-in interviews. We had 250 responses to the question of “Loop use in the previous month”; of these, 95 (38.0%) responses reported “some Loop use” over the previous month, and 155(62%) reported “no Loop use” over the previous month. A participant likely responded at more than 1 timepoint to this question, and therefore, these are not independent responses.

#### Acceptability

Perception of Loop’s acceptability [24] as agreeable, palatable, or satisfactory was explored in Phase 1 of implementation, which centered on program composition and workflows, and identification of a site champion who could facilitate buy-in and recruitment. Using an iterative consultative planning process, each site arrived at a decision to proceed or to not proceed with Loop. Among the sites we initially approached, one site did not proceed to Phases 2 and 3 due to competing priorities with the roll-out of another eHealth tool. Among the sites that proceeded to Phases 2 and 3, all participants perceived Loop, the implementation plan, and study procedures as acceptable. Perspectives on acceptability at midpoints and study end are presented in the monthly check-in and CFIR interview analyses below.

#### Appropriateness

Loop’s perceived fit, relevance, or compatibility for setting and gap in care is reported in the monthly check-in and CFIR analyses below.

#### Feasibility

The extent to which Loop was successfully used within a site was operationalized by recruitment and use statistics, reported in Tables 4 and 5. These data show that Loop was used in each of the participating sites to some extent. It is important to note, however, that sites were recruited within a research context, and site participants may have been motivated to use Loop for this reason.

### Barriers and Facilitators

#### Monthly Check-In Interviews

Monthly check-in interviews captured barriers and facilitators related to Loop use, ways in which Loop filled gaps in care, opportunity to use Loop, team composition and patterns of communication, Loop design and function, and overall satisfaction with Loop. Field notes, reported as excerpted first-person statements below, shed light on loop acceptability and appropriateness.

#### Loop Use

Among barriers to Loop use, iHCPs, patients, and caregivers reported that existing modes of communication, such as phone, in person, and email were commonly used for medical care needs. As such, participants did not perceive a relative advantage [15] to using Loop for team-based communication, and medical issues were not sufficiently complex to warrant the use of Loop.

It’s not necessary to add new people on Loop...We have a system that is working well... Typically, just calling the nurse practitioner and/or emailing the nurse at the hospital works. I see the value in the Loop system, but it’s not necessary for where I am at.

Fieldnote, Parent Caregiver, Site 5, Month 2

#### Gap in Care

Loop’s inclusion of patients in team communication was perceived to be facilitative to Loop use and was identified as fulfilling a gap in care.

There is a strong advantage to having the patient in the Loop and being privy to these conversations.

Fieldnote, iHCP, Site 2, Month 1

The iHCP quoted above expressed how communication is a challenge, even when programs are part of the same organization and located in the same building. The conventional transfer of information via consult notes does not address this gap. Additionally, iHCPs said that Loop addressed a collaboration gap across the health care team. Patients also expressed that collaboration, specifically, is critical but lacking in the care they experience.

The collaborative care element is key, this is what is missing from the patient’s experience of the health system…it’s a crucial gap that Loop could fill, it’s just getting people on board to use it. There needs to be communication between different providers and different sites. The lack of communication leads to care being incredibly fragmented.

Fieldnote, Patient, Site 6, month 6

Several participants perceived Loop as having potential to improve their medical care and to prevent the duplication of communication and services. In addition, Loop could provide a means to ask questions or provide updates that may not have been communicated during in-person visits.

I found that providers were ‘duplicating’ some of the same treatment issues and that maybe Loop could be useful for this.

Fieldnote, Caregiver, Site 1, Month 4

Everyone is on Loop so I am feeling better about not needing to double up with messages. Anyone on the Loop can prescribe if the patient needs something. Any person on the Loop can do the duty needed, which is 11 people on Loop. I find that at home, we have so many services going on that one thing gets mixed up and all of a sudden all the information is wrong and I get stressed out. As a result, I feel like I am not sure what’s going on. Loop could help.

Fieldnote, Caregiver, Site 5, Month 2

#### Opportunity to Use Loop

Participants across all sites frequently reported that no medical situation arose during the data collection period that prompted them to post a message in Loop. Patients were either medically stable or in remission and, therefore not requiring active treatment; or they were too sick to use Loop, admitted to hospital or a palliative care unit. Patient and caregiver Loop use was facilitated by instances when a specific situation arose, such as an emergency department visit, a need to coordinate an admission to long-term care, or to ask a question about symptoms. In other instances, a patient or caregiver used Loop to update the health care team, primarily about appointments they had scheduled.

#### Team Composition and Patterns of Communication

Although some users reported that partial teams could still be useful, assembling additional HCPs in a Patient’s Loop proved challenging. Participants frequently stated that unless the relevant team members were enrolled, Loop had limited usefulness.

Loop on the other hand is very simple and easy. The goal is to have one place for all the specialists; one place they can go to communicate. I feel that if you can’t get everyone to sign on, then it limits the usefulness of Loop.

Fieldnote, Caregiver, Site 6, month 1

Participant messages that were left unreciprocated also posed a barrier to Loop use.

After trying this and getting no response, I didn’t want to use Loop more because I didn't want to feel that I was badgering others.

Fieldnote, Patient, Site 6, month 6

Some iHCPs reported that they did not post messages unless patients posted first, perceiving the patients or caregivers as drivers of care-based communication. Of note, in at least one instance, an adolescent patient stated that he would not post messages in Loop unless the HCPs posted first. Patients and caregivers whose messages were reciprocated indicated that they were likely to use Loop again.

#### Design and Function

Loop’s user interface was generally considered to be simple and intuitive, and the asynchronous nature of the messaging useful for nonurgent messages, and thereby, likely to reduce burden. Nonetheless, patients and caregivers who had access to their hospital’s patient portal, which allows them to view reports and test results, found the portal met many informational needs, if not their communication needs. Some users expressed that integration of Loop into the patient portal would be helpful.

I really, really, like the simplicity of Loop’s design, and I feel that it is simple to access for those that might not be tech savvy.

Fieldnote, Patient, Site 1, month 2

There was some confusion among participants about Loop’s purpose and the types of messages that were appropriate to post.

I felt unsure about what concerns can be put on the system. Right now, I generally send emails to my HCPs regarding care plans. There are 12 members in my Loop, but no activity.

Fieldnote, Caregiver, Site 5, month 2

iHCPs commonly believed Loop should ideally be integrated into the hospital’s electronic medical record (EMR). Because Loop requires its own login and is not embedded in the EMR, its use was cumbersome and did not align with their existing workflow, particularly if only a few of their patients were using it.

#### Implementation Context

Given that research was the initiating context for the implementation endeavor, research team members played key roles in supporting implementation that would not be sustainable otherwise. Study staff helped users to register on Loop, explaining what tagging “attention to” someone means when posting a message, and clarifying what kinds of messages were appropriate to post. Study staff posted bimonthly messages with audit information such as the number of messages posted in the participant’s Patient Loop, number of messages in the most active Patient Loop across all sites during the same period, how to use Loop, and updates about study.

#### Workflow and Compatibility

iHCPs had pre-existing processes or workflows that were supported by administrative or other clinical staff. In some settings, clinical administrative staff or trainees were tasked with communicating with patients and other HCPs, which meant HCPs did not experience the back and forth “telephone tag” that is common when communicating with patients and other HCPs. This removed some of these inefficiencies in communication that we anticipated would be a stimulus for Loop use. Furthermore, some iHCPs reported that they would want an intermediary to function in a similar administrative or facilitative role within Loop.

Other workflows relied on the patient (or family) to initiate communication, as with the transfer of information between organizations. In this situation, Loop’s advantage in reducing the patient or caregiver’s responsibility for transmitting information from HCP to HCP was not realized.

The patient is very helpful in communicating for herself. For example, she acts as the focal point for communication, prints out test results, and updates for me and provides them at the beginning of a visit for me to review.

Fieldnote, iHCP, Site 1, month 3

#### Overall Satisfaction With Using Loop

Monthly check-in interviews provided feedback on participants’ satisfaction with Loop. Across all timepoints, 45 responses indicated users were “somewhat satisfied” to “very satisfied,” and 7 responses indicated users “somewhat dissatisfied” and “very dissatisfied.” Satisfaction feedback was only elicited if the participant had used the system in the previous month. We inferred that any interview that did not have a response for “satisfaction” or was coded as “unable to rate” had no Loop use. The denominator for the satisfaction question was 279 responses, and do not reflect independent responses because the same participants may have replied to this question at more than 1 timepoint.

#### COVID-19 Pandemic

The pandemic restrictions began in March 2020 in Canada and impacted recruitment and follow-up at Sites 4, 5, and 6. During this time, a parent caregiver would have liked to use Loop to check information about upcoming appointments, but at month 1, none of the additional HCPs had yet been assembled on their Loop. One iHCP reported loving the idea of Loop but felt it was difficult to build buy-in with other HCPs and patients during the pandemic. All contact with patients and other HCPs had shifted to virtual means and they found it hard to remember to talk about Loop. Another shift was that the pandemic led to removal of prior provincial restrictions on the use of alternative forms of communication and most care encounters became virtual. New billing codes for encounters by phone or videoconferencing were introduced. This change resulted in alternate methods of communication becoming incentivized and presented an unanticipated barrier to Loop use.

#### CFIR Interviews

CFIR interviews served to identify contextual barriers and facilitators according to this widely accepted determinant framework. CFIR comments were captured for each construct regarding its presence or absence in relation to supporting the implementation of Loop. Sites 2 and 6 had only 1 CFIR interview; sites 4 and 5 had 2 CFIR interviews; and sites 1 and 3 had 3 CFIR interviews. Notes for each site were summarized by construct by ES and RD and discussed with MB. Valence was rated for each construct by interview, but the mode could not be calculated for 2 sites where only 1 HCP was interviewed, and so valence is not reported numerically. Rather, coders reviewed the construct summaries by site and coded them as to whether they were perceived as supportive of implementation or not (yes/no), and as being present or absent in Loop implementation (+/-). Table 6 presents these findings: where codes were mixed, this is noted, and where constructs did not manifest in the interview, they are left blank.

**Table 6:** Salient CFIR^a^ constructs by site.

CFIR domains and constructs			Site 1	Site 2	Site 3	Site 4	Site 5	Site 6
Interviews, n			1	3	3	1	2	2
Message frequency quartiles (Hi, Med, Lo)^b^			Q3 (Hi)	Q3 (Hi)	Q3 (Hi)	Q1 (Lo)	Q2 (Med)	Q2 (Med)
**Intervention characteristics**								
	Intervention source		Yes (+)	Yes (+)	—	Yes (+)	Yes (+)	Yes (+)
	Evidence strength and quality		—	—	Mixed	Yes (+)	Yes (+)	Yes (+)
	Relative advantage		Yes (–)	Yes (+)	Mixed	Yes (–)	Yes (+)	Yes (+)
	Adaptability		Yes (–)	Yes (–)	Yes (–)	Yes (–)	Yes (–)	Yes (–)
	Complexity		Yes (–)	Yes (+)	Yes (+)	Yes (+)	Yes (+)	Yes (+)
**Outer setting**								
	Patient needs and resources		Yes (+)	Yes (+)	Mixed	Yes (+)	Yes (+)	Yes (+)
	Peer pressure		—	—	—	—	—	—
	Cosmopolitanism (no score)		—	—	—	—	Yes (+)	—
	External policies and incentives		Yes (+)	—	Yes (+)	—	Yes (+)	Yes (+)
**Inner setting**								
	Structural characteristics		Yes (–)	Yes (–)	Yes (–)	Yes (–)	Yes (–)	Mixed
	Networks and communications		—	—	Yes (–)	—	Yes (+)	Mixed
	Culture		Yes (+)	Yes (+)	Mixed	Yes (–)	Yes (+)	Yes (+)
**Implementation climate**								
	Tension for change		Yes (+)	Yes (+)	Mixed	—	Yes (–)	Yes (+)
		Compatibility	Yes (–)	Yes (–)	Yes (–)	Yes (–)	Yes (–)	Yes (–)
		Relative priority	—	Yes (–)	Mixed	—	Yes (–)	
		Organizational incentives and rewards	—	—	Yes (–)	—	—	Yes (–)
	Goals and feedback		—	—	Yes (–)	—	Yes (+)	—
	Learning climate		Yes (–)	Yes (+)	Mixed	Yes (+)	Mixed	Yes (+)
	Leadership engagement		Yes (–)	Yes (–)	Yes (–)	—	Yes (–)	
	Available resources		Yes (+)	Yes (+)	Yes (+)	Yes (+)	Yes (+)	Yes (+)
	Access to knowledge and information		Yes (+)	Yes (+)	Yes (+)	Yes (+)	Yes (+)	Yes (+)
**Characteristics of individuals**								
	Knowledge and beliefs about the intervention		Yes (+)	Yes (+)	Mixed	Yes (+)	Yes (+)	Yes (+)
	Self-efficacy		Yes (+)	Yes (+)	Yes (+)	Yes (+)	Yes (+)	Yes (+)
	Individual stage of change (no score)		—	—	—	Yes (+)	Yes (+)	—
	Individual identification with organization (no score)		—	Yes (+)	Mixed	Yes (+)	Yes (+)	—
	Other personal attributes		Yes (+)	Yes (+)	Yes (+)	Yes (+)	Yes (+)	Yes (+)
**Process**								
	Planning		Yes (+)	Yes (+)	Yes (+)	Yes (+)	Yes (+)	Mixed
	Opinion leaders		—	Yes (+)	Mixed	Yes (+)	—	Mixed
	Formally appointed internal implementation leaders		—	—	Yes (–)	Yes (+)	Mixed	—
	Champions		—	Yes (+)	Yes (–)	—	Yes (+)	—
	External change agents		—	—	Yes (–)	—	Mixed	Yes (–)
	Executing		—	—	Mixed	Yes (–)	Yes (+)	Mixed
	Reflecting and evaluating		Yes (+)	Yes (+)	Yes (–)	—	Yes (+)	Yes (+)
**Characteristics of recipients^c^**								
	Patient beliefs		Yes (+)	—	Mixed	Yes (+)	Yes (+)	Mixed
	Patient experience		Yes (+)	Yes (+)	—	—	Yes (+)	Yes (–)

^a^CFIR: Consolidated Framework for Implementation Research.

^b^See Table 4.

^c^Not original to CFIR.

Several CFIR constructs were perceived as supporting implementation and as having manifested in Loop implementation across most sites. These are annotated as “Yes (+)” in Table 6.

#### Intervention Characteristics (Intervention Source and Complexity)

Respondents were aware of and had positive regard for where Loop originated, and this was perceived to be supportive of its implementation. Loop was viewed as easy to use, which was also facilitative for its implementation.

#### Outer Setting Characteristics (External Policies and Incentives)

Respondents perceived the outer health system context as supportive of tools that could improve communication within the patient’s circle of care and saw this as supportive of Loop implementation.

#### Inner Setting Characteristics (Culture, Available Resources, Access to Knowledge, and Information)

Organizational culture was perceived as supportive of initiatives to implement evidence-based interventions such as Loop. Respondents felt they were well supported by the research team and had access to requisite knowledge and information about Loop in a way that supported implementation.

#### Characteristics of Individuals (Knowledge and Beliefs About the Intervention, Individual Identification With the Organization, Other Personal Attributes)

Respondents felt they were familiar with facts, truths, and principles related to Loop, perceived their organization was committed to evidence-based care, and possessed the requisite tolerance of ambiguity, intellectual ability, motivation, values, competence, capacity, and learning style.

#### Process (Planning, Reflecting, and Evaluating)

Respondents were aware of the plan in place to support Loop implementation and perceived this as facilitative. They also valued the opportunity to reflect on their experience and use of Loop during the monthly check-in interviews, although they were unsure as to how their reflections were used to inform ongoing implementation.

#### Characteristics of Recipients (Patient Beliefs and Patient Experience)

Respondents felt patients believed in the usefulness of Loop and that, for the most part, their experience of using Loop was positive.

Several CFIR constructs were perceived having the potential to support implementation generally but were perceived as absent with respect to Loop implementation across most sites—indicated as Yes (–) in Table 6.

#### Intervention Characteristics (Relative Advantage and Adaptability)

Respondents felt that although Loop had potential over alternative solutions, this relative advantage was not realized. They would have liked Loop to be adaptable to their workflow and environment, specifically with respect to its integration with the local EMR or patient portals.

#### Inner Setting Characteristics (Structural Characteristics, Tension for Change, Compatibility, Relative Priority, Organizational Incentives and Rewards, and Leadership Engagement)

With respect to the structural characteristics of the implementing organizations, respondents felt that Loop should ideally be integrated with the EMR they were already using. The lack of integration was perceived as a major barrier to Loop use as it meant users had to take several extra steps to enter and extract Loop information to put into the medical record.

Having more than one system to work with is always going to be very awkward.

CFIR interview, iHCP, Site 1

The lack of integration between Loop and EMRs also emerged as a barrier with respect to Loop’s compatibility with existing workflows. These workflows are dictated by and facilitated through the EMR; anything outside of the EMR is difficult to manage.

It’s already complicated by the fact that nurses who take care of the same patients, do not use the same record-keeping system as I do... so there is already additional work needed to fill this gap.

CFIR interview, iHCP, Site 1

Tension for change was low across all sites, given the perception that Loop was needed by only a small number of more complex patients. Respondents noted a lack of organizational incentives and rewards and limited involvement from organizational leadership. Several changes would be needed to implement Loop more effectively, including leadership engagement, use of reminders, and elimination of multiple passwords across various systems through integration with EMRs and portals.

#### Process (Formally Appointed Internal Implementation Leaders and External Change Agents)

Most of the constructs related to engaging others in the implementation process received mixed and largely negative ratings. Respondents commented on the limited influence of opinion leaders, champions, and external change agents.

We explored CFIR constructs found to be salient in this study relative to other studies in which CFIR was assessed by interview (see Table 7). Although 2 of the comparison studies [22,49] compared high and low implementing sites, 2 studies did not ([23] and this study). All 4 studies explored implementation in different contexts and for different interventions yet results for salient constructs are surprisingly similar in at least two or more of the studies, which suggest that several consistently robust constructs are commonly associated with implementation. Highly salient constructs across studies include relative advantage; patient needs and resources; external policies and incentives; tension for change; available resources; knowledge and beliefs about the intervention; and implementation planning. Salient to at least two studies were constructs of adaptability, complexity, structural characteristics, culture, compatibility, leadership engagement, access to information and knowledge, reflecting and evaluating, and beliefs of the health care recipient.

**Table 7:** Salient CFIR^a^ domains across studies.

CFIR domains and constructs			Damschroder and Lowery (2013) [22]^b^	Varsi et al (2015) [50]^b^	Barwick et al (2015) [23]^c^	This study (2021)^c^
**Intervention characteristics**						
	Intervention source		—	—	—	Yes (+)
	Evidence strength and quality		—	—	—	Yes (+)
	Relative advantage^d^		Yes	Yes	Yes	Yes (–)
	Trialability		—	Yes	—	—
	Adaptability^e^		—	—	Yes	Yes (–)
	Complexity^e^		—	—	Yes	Yes (+)
**Outer setting**						
	Patient needs and resources^d^		Yes	Yes	Yes	Yes (+)
	Cosmopolitanism		—	—	Yes	—
	External policies and incentives^d^		Yes	—	Yes	Yes (+)
**Inner setting**						
	Structural characteristics^e^		—	Yes	—	Yes (–)
	Networks & Communications		Yes	—	—	—
	Culture^e^		—	Yes	—	Yes (+)
	**Implementation climate**					
		Tension for change^d^	Yes	Yes	Yes	Yes (+)
		Compatibility^e^	—	Yes	—	Yes (–)
		Relative priority	Yes	Yes	—	—
		Goals and feedback	Yes	—	—	—
		Learning climate	Yes	—	—	—
	**Readiness for implementation**					
		Leadership engagement^e^	Yes	—	—	Yes (–)
		Available resources^d^	Yes	Yes	—	Yes (+)
		Access to information and knowledge^e^	—	—	Yes	Yes (+)
**Characteristics of individuals**						
	Knowledge and beliefs about the intervention^d^		—	Yes	Yes	Yes (+)
	Self-efficacy		—	—	—	Yes (+)
	Individual identification with organization		—	—	—	Yes (+)
	Other personal attributes		—	—	—	Yes (+)
**Process**						
	**Planning** ^d^		Yes	Yes	—	Yes (+)
		Planning for sustainability	—	—	Yes	—
		Opinion leaders	—	—	—	Yes (+)
	Formally appointed internal implementation leaders		—	Yes	—	—
	Champions		—	—	Yes	—
	Reflecting and evaluating^e^		Yes	—	—	Yes (+)
**Characteristics of intervention recipients**						
	Patient beliefs^e^		—	—	Yes	Yes (+)
	Patient experience		—	—	Yes	—

^a^CFIR: Consolidated Framework for Implementation Research.

^b^Related to constructs distinguishing between high and low implementers.

^c^Related to constructs identified as salient for implementation success: (+) construct was present; (–) construct was absent.

^d^Construct highly salient in more than 2 studies.

^e^Construct highly salient in at least two studies.

### Clinical Outcomes

The primary outcome of COC was the Picker COC for which higher scores denote better COC. At baseline, the COC mean (SD) was 48.62 (29.88) for 48 patients. At month 2, the mean (SD) was 53.95 (29.18) for 19 patients. At month 6, the mean (SD) was 60.71 (26.79) for 14 patients. The descriptive statistics for all the surveys at each timepoint are presented in Table 8. COC scores showed a significant (*P*<.001) mean change (SD) of 24.23 (SD 26.01) in the positive direction at month 6 from baseline; however, none of the other timepoints showed a significant change in COC score and the rates of incomplete data limit any inference (Table 9). Similarly, an exploratory repeated measures analysis using generalized estimation method with autoregressive (AR-1) covariance structure for adjusting for repeated measures within patients with the outcome of COC, controlling for CCRQ and number of messages per Patient Loop, yielded no significant associations. We were unable to draw conclusions from the survey data with regard to appropriateness.

**Table 8:** Patient Surveys: Summary Descriptive Statistics.

Variable		Baseline, n/mean (SD)	M2, n/mean (SD)	M4, n/mean (SD)	M6, n/mean (SD)	M8, n/mean (SD)
COC^a^		48/48.62 (29.88)	19/53.95(29.18)	13/52.88 (24.02)	14/60.71 (26.79)	5/55.00 (22.71)
SSpedi^b^		6/22.50(12)	4/23.50 (4.12)	—	—	—
**ESAS^c^**						
	Physical	40/17.02 (11.76)	16/16.25 (10.61)	13/16.54 (11.54)	14/17.71 (13.41)	5/17.00 (14.58)
	Emotional	40/6.25 (5.93)	16/6.50 (6.34)	13/6.31 (6.34)	14/6.57 (6.93)	5/8.60 (7.02)
	Well-being	40/4.33 (2.57)	16/4.44 (2.61)	13/4.62 (2.72)	14/4.21 (3.07)	5/4.20 (1.30)
	Total symptom score	40/27.60 (17.65)	16/27.19 (17.21)	13/27.46 (18.80)	14/28.50 (22.60)	5/29.80 (20.29)
CCRQ^d^: CPDG^e^		44/81.25 (18.05)	19/83.77 (15.94)	12/77.15 (8.85)	13/82.40 (27.09)	5/95.00 (5.43)

^a^COC: Picker Ambulatory Cancer Care Scale, Continuity, and Coordination subscale.

^b^SSpedi: Symptom Screening in Pediatrics Tool.

^c^ESAS: Edmonton Symptom Assessment Scale.

^d^CCRQ: Client-Centered Rehabilitation Questionnaire.

^e^CPDG: Client Participation in Decision-making and Goal setting domain of CCRQ.

**Table 9:** Comparing patient baseline and follow-up COC^a^ measurements.

Timepoint	N	Mean change in score (SD)	Comparison timepoints	*P*-value
M2	18	10.97 (36.76)	M2 to baseline	.246
M4	13	10.00 (41.50)	M4 to baseline	.402
M6	13	24.23 (26.01)	M6 to baseline	.006
M8	5	18.50 (20.36)	M8 to baseline	.112

^a^COC: Picker Ambulatory Cancer Care Scale, Continuity, and Coordination subscale.

## Discussion

### Principal Findings

This hybrid type II mixed methods implementation study found that gaps in communication and collaboration persist. In the absence of a shared clinical communication tool, health care providers have increasingly adopted email and texting with patients and caregivers over the last decade, and this virtualization has been accelerated by the COVID-19 pandemic [50-52]. Although participants acknowledged these forms of communication have their disadvantages and they could see potential in using Loop, this relative advantage was not realized largely due to the lack of Loop integration with existing health information systems (compatibility). There was a range of message frequency observed, with sites #1, #2 and #3 showing relatively more use than the others. These differences were not reflective of different construct profiles at the sites. Participants who used Loop were mostly satisfied. As in our previous study, patients were more likely than HCPs to initiate communication on Loop [8]. The implementation of Loop was done in the context of research, rather than as an organizational change initiative, and was therefore constrained in scope and time. The lack of broad organizational engagement, relative priority, and tension for change in the implementing organizations further hampered Loop’s implementation. We were unable to make inferences about Loop’s clinical effectiveness due to insufficient follow-up and survey data.

### Implementation Effectiveness

In the context of this research implementation of Loop, our approach fell short in several ways and effective implementation remained elusive. Several studies have demonstrated that engaging local champions who sustain commitment and garner organizational support facilitates successful practice change [53-56]. Although we identified committed site champions, their role was informal. Engagement efforts largely fell to research staff and we did not seek to establish broad organizational support for this time-delimited implementation endeavor. This level of engagement was insufficient to influence greater adoption of Loop; however, sample size limits the conclusions we can draw. Moreover, it is likely that the lack of Loop compatibility and nonintegration with local EMRs would have proven to be a nonmodifiable barrier to implementation despite more active engagement.

Phase 1 activities and CFIR interviews affirmed that Loop was deemed acceptable at all sites. As in our previous pilot RCT [8], we demonstrated the feasibility of implementing Loop operationally, while once again experiencing challenges in enrolling additional HCPs, and finding the optimal patient and health care context (opportunity) to show the value of team-based communication.

Lack of compatibility and relative advantage were key implementation barriers in this study. Successful implementation of a novel tool, particularly one that disrupts [57] existing workflows, requires finding the optimal context and patient population to achieve early gains. HCPs frequently expressed that Loop ought to be integrated with the EMR used for charting. Indirect integration or workarounds that involved exporting messages as PDF and uploading them into the EMR were too cumbersome. Adding to this technological conundrum is that, in Ontario, as in many global jurisdictions, care teams straddle multiple EMRs even within the same organization. Many organizations must revert to custom nonscalable models for third-party tool integration. Although the landscape is shifting—for example, the 21st Century Cures legislation in the United States promotes greater standards adoption including HL7 FHIR [58]—this is far from the status quo in Canada. Furthermore, communication standards, as one might use for Loop, are not included in these health technologies, which are focused on the exchange of discrete data such as laboratory results, medications, or documents.

Some organizations have launched patient portals, but these too differ from one organization to another, perpetuating information silos. Of note, 2-way communication is not a standard feature of all patient portals. Patients and caregivers who had access to a patient portal felt that Loop would be more useful if it were integrated with their patient portal. This integration makes sense given that in both our Loop feasibility RCT and in this study, patients and caregivers were more often the drivers of communication. However, the prevailing institutionally tethered models may limit the addition of crucial external team members. Moreover, while patient portals with messaging capability have been shown to improve patient satisfaction and increase the “meaningful use” of data, few studies show that they improve health outcomes [59]. The nature of portals is changing with the emergence of institutionally agnostic commercial models such as Apple’s Health Records entering the space, which may offer an opportunity for communication in the patient’s circle of care.

Although participants perceived Loop’s relative advantage over existing communication channels, this advantage was not actualized, presenting a key barrier [60-62] that would have been difficult to address using deimplementation strategies [63]. Patients, caregivers, and HCPs are reluctant to use yet another tool for communication. They would rather leverage Loop-like functionality (security, organized storage, and retrieval of clinical communication) in tools they already use to communicate in the nonhealth care aspects of their lives. However, none of the email platforms commonly in use provide these functions. Moving to a new communication tool will require realizing greater relative advantage, because efforts to deimplement commonly used means of communication are unlikely to work.

The Collaboration Space Model proposed by Eikey et al [5] outlines the following processes related to collaboration: workflow, communication, and information exchange [5]. The model also proposes 2 outcomes related to collaboration: maintaining awareness and establishing common ground. Applying this model to our study, we observed that participants who used Loop expressed that Loop had a mostly positive impact on the processes of communication and information exchange, and the outcome of maintaining awareness. Of note, a number of participants did not use Loop. The predominant perspectives were that Loop was disruptive of existing workflow. Although there were instances of coordination and cooperation occurring in Patient Loops, we did not observe an impact on the higher-order collaboration outcome of establishing common ground. In addition to the barriers already discussed, our study was not designed to focus on the requirements for collaborative care in the context of a single site. In the absence of being able to effect change in organizational processes and structures, the adoption of Loop was hampered.

### Clinical Effectiveness

Pooled survey data across all sites suggested an isolated improvement in continuity and coordination of care from baseline to month 6 but not at other timepoints. This should be interpreted with caution because fewer than 50% of participants completed survey responses beyond baseline.

### Health System and Policy Environment

Since the start of the Loop research program in 2012, we have navigated an ever-shifting landscape. Ontario’s health care environment has experienced major policy changes in the past 3 years, including the restructuring of regional health authorities (Local Health Integration Networks) into a new model of networks of care called Ontario Health Teams. While this new organizational structure may hold promise for the scaling of eHealth solutions in the future, the transitional period has resulted in deferred decision making. Although we cannot be certain, these system changes may have impacted the progress of our work in terms of informing policy and partnering with provincial organizations for the sustainability of Loop beyond the research program.

A relevant policy shift emerging from the COVID-19 pandemic was the necessity of providing care outside the in-person visit, and permission to communicate and bill for care using phone and video. The perspectives that emerged during our study suggest that the net effect of this system shift was unfavorable for Loop use.

A larger structural issue is related to models of compensation for physicians, nurses, and other HCPs that incentivize in-person or synchronous virtual encounters. Physician fee for service, capitated, and alternate payment plans likely impact the use of Loop. Notably a recent study found very high rates of patient and provider desire to engage in asynchronous messaging preferentially versus using synchronous video or phone in a primary care [64], capitated setting where the family physician and patients accrue benefits without penalties. For physicians working in the fee for service model, including most specialists, there are no billing codes for asynchronous communication. Similarly, there are different models of compensation for nurses. As an example, in Ontario, home care nurses are required to do a certain number of face-to-face visits under one model. The nurses who participated in our study were salaried. All iHCPs were affiliated with academic organizations, likely influencing their willingness to participate in research. We were unable to observe a difference in Loop adoption across different forms of HCP compensation.

Some health system changes were facilitative for Loop. In 2019, the College of Physicians and Surgeons of Ontario disseminated 4 interrelated policies for improving COC in Ontario [65]. While this action raised COC as a priority, it focused mainly on ensuring that physicians provide appropriate options for after-hours care and reliable processes for effective transitions in care. It is possible that compliance with these recommendations will advantage use of asynchronous team-based communication. However, these recommendations have been met with resistance from various stakeholders and their implementation and long-term impact remain uncertain.

### Future Research, Health Equity, and Accessibility in eHealth Tools

The challenges encountered in this study are common in studies of eHealth tools, and more generally of implementing complex interventions in complex settings. However, a compelling finding was that Loop disrupted workflows and workarounds that have developed in the absence of standardized tools for communication. Loop could not transcend established communication modalities despite their inability to enable team communication and collaboration. Future research could specifically focus on a health care region, integrate Loop with existing eHealth tools, pilot a compensation structure for asynchronous communication, deimplement or re-design optimal communication workflows from the ground up, and support requirements or conditions for collaboration to occur. In addition, future research should examine the impact of eHealth implementation on health disparities, which have been shown to increase with the introduction of patient–provider messaging tools [66]. Despite the considerable and seemingly intractable barriers identified in this study, the trend toward more digital communication in health care is inevitable and it is likely that an interoperable system of communication and documentation will emerge in time. It is important that tools such as Loop be available and accessible to all, so that health inequities are not further magnified.

### Limitations

The research context of this implementation endeavor likely introduced bias insofar as health care providers and patients were possibly more inclined to use Loop to fulfill their commitment as research participants. In addition to participant, team, and site selection bias, we acknowledge the possible researcher bias: the main reviewer (AH) for the qualitative check-in analysis is also the lead for the Loop research program.

Recruitment may have been impacted by issues of equity and access. Although smartphone and internet penetration are rising in Ontario, increasing from 81.4% in 2010 [67] to 92.2% in 2018 [68] with 89.1% of Ontarians reporting having a smartphone for personal use in 2018 [69], there are persisting disparities in access among the rural and marginally housed [70,71]. Given that internet access is a core component of Loop, access is not equitable to all potential patients.

Beyond the anticipated patient attrition rates, there was an unexpectedly low completion rate for clinical survey data beyond baseline. This limited the inferences that could be made about Loop’s clinical effectiveness. Participants from whom we were unable to collect data may have been more likely to talk about barriers to using Loop, and this too may have skewed our qualitative analyses. The challenges in recruiting participants at each of the sites limited our ability to mitigate attrition by over-recruiting within the timelines of the study. As in the previous study, recruiting additional HCPs to participate on the patient’s Loop team remained difficult and limited the use of Loop among those already enrolled.

The COVID-19 pandemic resulted in dramatic changes to the health care system in Ontario starting in March 2020, impacting recruitment, relative advantage in light of encouraged use of phone and email and permissible billing, and data collection in the follow-up periods (Phases 2 and 3) at Sites 4, 5, and 6. In the first 2 months of the pandemic, all research study recruitment in Ontario was paused unless deemed essential to the health of the participant or relevant to the pandemic. Although the impact was felt on many levels including study staff workflow, the main challenge was that the attention of many HCPs and health care leaders was focused on planning for the health care challenges posed by the pandemic.

The study was limited in being able to support the collaborative requirements at each site. Therefore, a weakness of the study was its focus on Loop without being able to substantively support the structures and processes that would have allowed us to impact collaboration.

### Conclusions

This study highlighted the importance of system and organizational context and several key determinants of effective implementation. From the start of the Loop program of research, regulatory guidelines have restricted the use of email and text due to privacy concerns and created data silos within organizations. Despite these restrictions and in the absence of other practical tools for communication, there has been a steady increase in the use of email, text, and other forms of messaging to provide the care that patients need. The COVID-19 pandemic shed light on the essential components of that care. Delivering care became the priority and the regulatory guidelines became pragmatic. The health system learned that a considerable proportion of the care HCPs provide in person can be provided virtually, by video or phone, if the HCP is compensated for this mode of service delivery. If a rational approach to the regulatory framework continues and health leadership prioritizes an integrated digital infrastructure, we may yet achieve the goal of care being provided by the right person, in the right place, at the right time, and with the right tools.

Key facilitative factors again show themselves to be essential for effective implementation. Perceived relative advantage only goes so far, and this study demonstrates, yet again, that compatibility, relative advantage, tension for change, and engagement are essential implementation components that must be realized.

Fundamental structural challenges remain for the implementation and scaling of a shared system of asynchronous communication, including digital integration and a fee structure for compensation. If a new hybrid model of care emerges from the pandemic, it is likely to de-emphasize in-person encounters between patients and HCPs, disrupt existing workflows, and allow the intentional design of new pathways for care that prioritize team communication, access, and COC. Until these changes manifest, effective implementation of Loop and similar communication platforms will continue to be elusive.

## Appendix

Multimedia Appendix 1 Standards for Reporting Implementation Studies: the StaRI checklist for completion.

Multimedia Appendix 2 Semi-structured interview for Health-Care Providers based on the Consolidated Framework for Implementation Research.

## Abbreviations

ACCI: Age-Adjusted Charlson Comorbidity Index
CCRQ: Client-Centered Rehabilitation Questionnaire
CPDG: Client Participation in Decision Making domain of CCRQ
CFIR: Consolidated Framework for Implementation Research
COC: continuity of care
ECOG: Eastern Cooperative Oncology Group
EMR: electronic medical record
HCP: health care provider
iHCP: initiating health care provider
QIF: Quality Improvement Framework
RCT: randomized controlled trial
