# Supplementary material for: A Clinical Communication Tool (Loop) for Team-Based Care in Pediatric and Adult Care Settings: Hybrid Mixed Methods Implementation Study
Source: J Med Internet Res. 2021 Mar 3;23(3):e25505. doi: 10.2196/25505 (PMC8294640; doi:10.2196/25505)
Supplement: Multimedia Appendix 2 [file jmir_v23i3e25505_app2.pdf]

Appendix 2: Semi-structured interview for Health-Care Providers based on the Consolidated Framework for Implementation Research (CFIR; adapted from Damschroder & Lowery, 2013)

"In order to be sure what you say here today is accurate we would like to audio-record these monthly sessions which may then be transcribed. If we transcribe these recordings, any identifying information would be replaced with pseudonyms. We will store these recordings, along with all study data, for 10 years. Agreeing to having this session recorded is completely voluntary, will not affect your clinical care, and will not be shared with anyone else beyond the research team. Do you consent to having this session recorded (yes/no)."

If yes: *"Thank you, I will turn the audio recorder on now, and just ask that you repeat that you consent to having this session recorded."*

CIFR Construct	Interview Question
**GENERAL (Non CFIR)**	
	1. Please state your name and organization, for the tape<br>2. What was your role in the Loop implementation study?<br>*a. A physician on the project / an implementation lead, or a champion?*
**OUTER SETTING**	
**Patient needs & resources**	1. To what extent is Loop needed by patients and families?<br>*a. Why or why not?*<br>2. To what extent was this need taken into account in planning and implementation?<br>3. To what extent do you think patients and families perceive Loop as important?
**Peer pressure**	1. Are you aware of other organizations or teams that are implementing Loop? Or other similar patient communication tools?<br>2. How do you think the fact that other organizations may or may not be implementing Loop affects your own support for Loop?<br>3. How does using Loop provide any type of advantage for your organization?<br>*a. What about for you?*
**Cosmopolitanism**	1. To what extend do you network professionally with other individuals who are outside of your setting?<br>a. How have your professional affiliations influenced your work?<br>b. Are any of the individuals you network with implementing Loop?
**External policy and incentives**	1. To what extent were there local or provincial pressures to improve patient communication in your work setting?<br>*a. Did any recent health policies that influenced the implementation of Loop?*<br>2. Outside of your organization, who else is advocating or pressuring for improved patient communication?
**PROCESS**	
**Planning**	Now I would like to hear about the process you went through to implement Loop. Later in the interview we will talk about some parts in more detail.

		1. For now though please describe how Loop was implemented in your organization, to the best of your recollection?<br>a. *Who was involved in the planning process?*<br>i. *Do you think the appropriate people were involved in planning the implementation process?*<br>1. *Was anyone left out?*<br>b. *Can you describe the planning that was done to get Loop implemented?*<br>2. Where are you now in the process of implementing Loop: not at all implemented, fully implemented, halfway?
**Engaging**		
	Opinion leaders	1. Who were the key influential people that were instrumental in implementing Loop in your setting?<br>a. *How did they influence others to use Loop? How did they influence implementation?*<br>b. *Any opinion leaders?*<br>c. *Any key stakeholders?*<br>2. What steps were taken to encourage individuals to commit to using Loop?<br>a. *Which individuals will you target?*<br>b. *How will you approach them?*<br>c. *What information will you give them?*<br>d. *How frequently and how will you communicate with them?*<br>3. How did you communicate to the patients and families about Loop?
	Formally appointed internal implementation leaders	*1.* How was the decision made to implement Loop?<br>a. *Who participated in that decision?*<br>b. *Did you?*<br>2. Who was appointed to lead Loop implementation in your setting?<br>a. *Was this a formal or informal role?*<br>b. *Who else was involved?*
	Champions	1. Apart from the leaders you've mentioned, who would you say were champions of implementing Loop? That is, who worked to support implementation of Loop in important ways?<br>a. *Did they help persuade people who may have initially been indifferent or resistant to the program?*<br>b. *How so?*
	External change agents	1. Did anyone from an external organization formally influence or facilitate the implementation of Loop in your setting?
**Executing**		1. Has Loop been implemented according to the implementation plan that was shared with you?<br>2. To what extent do you think the implementation plan was collaboratively developed with your setting?
**Reflecting & evaluating**		1. Do you receive any regular feedback on Loop use?

		a. If YES: What kind of feedback do you receive?<br>*b. How is the feedback used?*<br>c. *Is it discussed with others?*
**INNER SETTING**		
	**Structural characteristics**	1. Outside of this research project, what kind of infrastructure would be needed to use and maintain Loop in your setting?<br>o *Changes in scope of practice? Changes in formal policies? Changes in information systems or electronic records systems? Other?*
	**Networks and communications**	1. What kinds of communications took place to support Loop?<br>*a. How were they important?*<br>2. What other services or organizations in the community did you work with to implement Loop if any?<br>3. What other departments or individuals within your organization did you work with to implement Loop?
	**Culture**	1. How would you describe the culture of your organization?<br>2. How do you think this culture has affected the implementation of Loop?<br>3. To what extent are new ideas embraced in your organization?<br>*a. Can you think of an example of this?*
	**Implementation climate**	*This question is likely to uncover topics to explore more within other sub-constructs, but be attentive to other themes that may not be included in your assessment.*<br><br>1. What was the general level of receptivity in your organization for implementing Loop?<br>o *Please describe*
	Tension for change	1. Is there a strong need for Loop?<br>a. Why or why not?<br>b. Do you think Loop meets the needs of patients and HCPs in your organization?<br>2. To what extent do you feel patients value Loop?
	Compatibility	1. How well does Loop fit with existing work flows and practices in your setting?<br>2. Did Loop replace or complement a current practice for improving team communication?<br>*a. If yes, were you able to successfully integrate Loop into existing operations and practices?*<br>*i. How was this done?*<br>b. *Did the implementation of Loop cause any issues?*
	Relative priority	1. When you first began to use Loop, were there other implementations or new initiatives taking place at the same time?<br>*a. What were these other initiatives?*<br>2. Were any of these other initiatives related to the improving team communication?<br>*a. If so, please explain?*<br>3. How do you typically juggle competing priorities related to new interventions like Loop?<br>4. Among the other new initiatives happening in your setting, which one has the highest priority?<br>*a. How did (priority mentioned above) come to have priority?*<br>*b. From your perspective, who decides the priority?*

			5. Do you feel that you have enough time to dedicate to learning about and using Loop?<br>*a. Have you had to put off other responsibilities you have to make time for learning about and using Loop?*<br>6. Does using Loop take away or interfere with other things that you need to get done in a typical day? *Please describe.*
		Organizational incentives and rewards	1. Can you think of any incentives that influenced the implementation of Loop (for HCPs? For patients?)<br>*a. If YES – how did they influence program implementation?*<br>b. *If NO, have you or others involved in the program received any recognition?*<br>2. Was using Loop acknowledged – *your efforts to use it* - in some way?
		Goals and feedback	1. Did your organization set any goals for Loop implementation, as in number of teams or patients?<br>2. Did your organization monitor how Loop was being used, formally or informally?<br>*3.* Did you receive any feed?back in any way about your use of Loop?<br>*a. From whom?*<br>4. How does implementation of Loop align with other organizational goals?
		Learning climate	1. Has your organization taken on any other recent implementation initiatives that have included you?<br>*a. What was it?*<br>*b. What made it successful or not?*<br>*c. What was your role?*<br>*d. Were people happy with the outcome?*<br>*e. Were leaders involved? How so?*<br>2. Do you feel you have the time and energy to take on quality improvement initiatives?
	**Readiness for implementation**		
		Leadership engagement	1. What level of involvement did top management at your organization have with the implementation of Loop?<br>*a. What kind of support did they give you? (specific examples)*<br>*b. Did they create any barriers for Loop implementation?*<br>2. What kind of support or actions can you expect from leaders in your organization to help make implementation successful?<br>*a. Who are these leaders? How do attitudes of different leaders vary?*<br>*b. Do they know about the intention to implement the intervention?*<br>*c. What kind of support can you expect going forward? Can you provide specific examples?*<br>d. *What types of barriers might they create?*
		Available resources	1. What kind of resources and external support did you receive during Loop implementation?<br>*a. Were there resources or support that would have helped you implement Loop that you didn’t*

			receive?<br>*b. What support is there for coaching, problem-solving, training?*<br>*c. Do you have adequate communications with Loop staff? Externally? Internally?*<br>*d. Do you feel adequately supported in using the tool?*<br>*e. Do Loop materials support your use of the tool?*<br>2. Did you have any resource constraints while getting Loop implemented? (Inadequate staffing? Inadequate time? Competing priorities?)<br>a. *If YES: what were they and how did that make a difference?*
		Access to knowledge and information	<u>TRAINING</u><br>1. What kind of training did you get for Loop?<br>2. Did you feel prepared to use Loop as a result?<br>3. Who do you go to for questions about Loop?
**INTERVENTION CHARACTERISTICS**			
	**Intervention source**		1. What's your understanding of who developed the Loop intervention and why it's being implemented here?
	**Evidence strength and quality**		1. What kind of information or evidence are you aware of that shows whether or not the intervention will work in your setting?
	**Relative advantage**		1. What kinds of programs/ activities for improving team communication did you already have?<br>*a. Do these other programs still exist?*<br>*b. Do you perceive Loop as being a better alternative?*<br>*c. Why or why not?*
	**Adaptability**		1. What kinds of changes or alterations do you think you will need to make to the intervention so it will work effectively in your setting?
Trialability			*Not addressed in this interview*
	**Complexity**		1. How complicated was it to use LOOP?
	**Design quality & packaging**		1. What materials or supports (note: technical or coaching via IP or phone) were available for facilitating the implementation of Loop? (Prompt: do you remember receiving a brochure or handout for Loop; demo?)<br>2. How well do these materials or supports facilitate the implementation process? Please describe.<br>3. How were these materials or supports helpful?<br>4. What other materials or supports would have been helpful to facilitate the implementation of the tool?
	Cost		1. What costs do you think were incurred by your setting to implement this intervention?<br>a. *Prompt: We brought you Loop courtesy of the research study, but did it involve extra activities on your time or extra resources?*
**CHARACTERISTICS OF INDIVIDUAL HCPS**			

	**Knowledge and beliefs about the intervention**	1. How important do you think team based communication is?<br>a. *Do you think Loop is the right solution to it?*
	**Self-efficacy**	1. How confident do you feel in using Loop?<br>a. *Why?*
	**Individual stage of change**	1. How prepared were you to start using Loop, when you began?
	**Individual identification with organization**	1. To what extent do you think how you identify and feel engaged with your organization make you more or less likely to adopt new interventions such as Loop?
	**Other personal attributes**	1. Did Loop's implementation align with your preferred learning style?<br>2. How motivated were you to use Loop?<br>3. How well do you think you used Loop?
**CHARACTERISTICS OF RECIPIENTS / PATIENTS**		
	**Patient beliefs**	1. In what way did Loop appeal to patients?<br>a. *What did your patients think of Loop?*<br>b. *Why do you think this?*
	**Patient experience**	1. What was the patient/family experience in using Loop?<br>a. *Prompt: How easy or difficult was it to use Loop?*
**DEFINING SUCCESS**		
	**Success**	1. How successful do you think Loop was at your site?<br>a. *Please describe*<br>2. If you had the option, would you recommend continuing the use of Loop (in the healthcare system)? What kind of impact did it have on patients and families?<br>3. If there was a way for you to receive infrastructure support for Loop, would you want to continue using it? (*Note: Infrastructure support includes formal agreements, data ownership, management of authentication and permissions*)
